# Pazopanib combined with doxorubicin and cisplatin in recurrent osteosarcoma: A retrospective cohort study

**DOI:** 10.1097/MD.0000000000046576

**Published:** 2026-01-09

**Authors:** Wei-Luo Cai, Mo Cheng, Zheng-Wang Sun, Meng Fang, Wang-Jun Yan, Xiong-Sheng Chen, Gen-Long Jiao

**Affiliations:** a Department of Spine Surgery, The First Affiliated Hospital, Jinan University, Guangzhou, China; b Department of Musculoskeletal Surgery, Fudan University Shanghai Cancer Center, Shanghai, China; c Department of Orthopedics, Shanghai Sixth People’s Hospital Affiliated to Shanghai Jiao Tong University School of Medicine, Shanghai, China; d Department of Orthopedic Surgery/Dongguan Key Laboratory of Central Nervous System Injury and Repair, The Sixth Affiliated Hospital of Jinan University, Dongguan, China.

**Keywords:** cisplatin, doxorubicin, osteosarcoma, overall survival, pazopanib, progression-free survival, recurrence

## Abstract

Treatment options for recurrent osteosarcoma remain limited, and prognosis is poor. Pazopanib, a multi-targeted tyrosine kinase inhibitor, has shown activity in various sarcomas. This study evaluated the efficacy and safety of pazopanib combined with doxorubicin and cisplatin (Paz + AP regimen) in patients with recurrent osteosarcoma and analyzed its impact on survival. This single-center retrospective cohort study included recurrent osteosarcoma patients treated between February 2022 and February 2023. Propensity score matching was used to balance baseline characteristics. A total of 100 patients were analyzed: 50 received the Paz + AP regimen, and 50 received conventional chemotherapy (AP regimen). Treatment efficacy was assessed using response evaluation criteria in solid tumors (RECIST 1.1) criteria. The primary endpoint was progression-free survival (PFS); secondary endpoints included overall survival (OS), objective response rate, disease control rate, safety, and treatment compliance. Baseline characteristics were well balanced between groups (*P* > .05). The Paz + AP group achieved a higher disease control rate than the control group (62.0% vs 42.0%, *P* = .046), whereas the difference in objective response rate was not significant (26.0% vs 14.0%, *P* = .13). Median PFS was significantly longer in the Paz + AP group than in controls (5.1 vs 2.2 months, *P* = .04). Although the median OS was slightly shorter in the Paz + AP group (8.0 vs 9.6 months), its survival curve declined more slowly after 12 months, showing a significant difference (*P* = .03). The incidence of adverse events was comparable (92.0% vs 88.0%), though hypertension (28.0% vs 10.0%, *P* = .03) and hand-foot syndrome (22.0% vs 4.0%, *P* = .01) were more frequent with pazopanib. No differences were observed in grade ≥3 or serious adverse events. Subgroup analysis indicated greater PFS benefits among patients with lung metastasis, recurrence interval ≥6 months, Eastern Cooperative Oncology Group (ECOG 0–1), and those receiving full-dose pazopanib. The Paz + AP regimen significantly prolonged PFS and improved disease control in recurrent osteosarcoma, with manageable toxicity and good compliance. Although OS improvement was limited, the regimen showed potential clinical value, particularly for patients with favorable performance status and longer recurrence intervals. Prospective studies are warranted to confirm these findings.

## 1. Introduction

Osteosarcoma is one of the most common primary malignant bone tumors, predominantly affecting adolescents and young adults, and is characterized by high malignancy and early metastatic potential.^[1,2]^ With the introduction of neoadjuvant chemotherapy and advances in surgical resection techniques, the 5-year survival rate for patients with localized osteosarcoma has improved to 60% to 70%. However, once recurrence or distant metastasis occurs – particularly pulmonary metastasis – the prognosis remains dismal, with a median overall survival (OS) generally <12 months and very limited effective therapeutic options.^[3,4]^

At present, there is no universally accepted standard treatment for recurrent osteosarcoma. Management primarily relies on chemotherapy regimens containing doxorubicin and cisplatin, such as the AP or MAP protocols.^[5]^ Nonetheless, patients with recurrent disease often exhibit markedly reduced sensitivity to conventional chemotherapy, with objective response rates (ORRs) typically below 20% and limited disease control.^[6]^ Against this background, exploring novel therapeutic strategies, particularly combination regimens, has become a focus of clinical and translational research.

In recent years, the emergence of molecular targeted therapy has provided new perspectives for systemic treatment of osteosarcoma. Angiogenesis plays a pivotal role in the progression and metastasis of osteosarcoma, making VEGF pathway inhibitors a subject of extensive investigation.^[7,8]^ Pazopanib, an oral multi-target tyrosine kinase inhibitor that inhibits vascular endothelial growth factor receptor, PDGFR, and c-KIT signaling, has demonstrated efficacy in delaying disease progression in a phase III trial for advanced soft tissue sarcoma, leading to FDA approval for unresectable or metastatic cases.^[9,10]^ However, studies of pazopanib in osteosarcoma remain limited, mostly consisting of small phase II trials or retrospective analyses. These suggest that pazopanib may achieve disease stabilization in some patients, though overall ORRs are low and the efficacy of monotherapy appears limited.^[11]^

Some scholars have proposed that the combination of tyrosine kinase inhibitors with chemotherapy may exert synergistic effects. On the one hand, vascular normalization can improve the tumor microenvironment and enhance the delivery of chemotherapeutic agents^[12]^; on the other hand, chemotherapy may induce stress responses in tumor cells, thereby increasing their sensitivity to anti-angiogenic therapy.^[13]^ However, evidence regarding the use of pazopanib in combination with chemotherapy for osteosarcoma remains very limited, with a lack of high-quality clinical data to support its application.

Based on this rationale, we retrospectively collected clinical data from patients with recurrent osteosarcoma treated at our institution and compared the efficacy and safety of pazopanib combined with doxorubicin and cisplatin (Paz + AP regimen) versus chemotherapy alone (AP regimen). Propensity score matching (PSM) was employed to minimize baseline differences between groups. The study primarily evaluated ORR, disease control rate (DCR), progression-free survival (PFS), OS, and safety profiles. Additionally, subgroup analyses were conducted to explore potential benefits across different patient populations. The aim of this study was to provide new clinical evidence for the treatment of recurrent osteosarcoma and to inform the design of future prospective clinical trials.

## 2. Materials and methods

### 2.1. Study design

This study was approved by the Ethics Committee of The First Affiliated Hospital of Jinan University. This was a single-center retrospective cohort study. We retrieved and collected clinical data of patients diagnosed with recurrent osteosarcoma who received treatment at our institution between February 2022 and February 2023. According to the treatment regimens administered after recurrence, patients were divided into 2 groups: Exposure group: received pazopanib in combination with doxorubicin and cisplatin. Control group: received conventional chemotherapy regimens without pazopanib, primarily doxorubicin plus cisplatin.

To minimize the impact of baseline differences between groups, we applied PSM. Covariates included in the propensity score model were age, sex, Eastern Cooperative Oncology Group (ECOG) performance status, site of recurrence, prior treatment regimens, and interval from recurrence to re-treatment. A 1:1 matching ratio with a caliper width of 0.2 was adopted. After matching, a total of 100 patients were included in the final analysis, with 50 patients in each group.

### 2.2. Inclusion and exclusion criteria

Inclusion criteria: Pathologically confirmed diagnosis of osteosarcoma. Prior receipt of standard first-line treatment (including neoadjuvant chemotherapy and surgery), followed by local recurrence or distant metastasis. Treatment after recurrence with either pazopanib combined with doxorubicin and cisplatin, or conventional chemotherapy without pazopanib (primarily doxorubicin plus cisplatin). Completion of at least 2 treatment cycles, with available radiological assessments and complete clinical follow-up data allowing efficacy and survival analysis. Age ≥18 years, ECOG performance status ≤2. Adequate cardiac, hepatic, and renal function sufficient to tolerate chemotherapy and targeted therapy.

Exclusion criteria: Patients who received pazopanib combined with chemotherapy at the time of initial diagnosis. Presence of other primary malignancies or active severe infections. Incomplete clinical records or follow-up data precluding evaluation of efficacy and survival. Participation in other clinical trials or concurrent treatment with novel investigational anticancer agents. Inability to complete the planned treatment regimen due to severe cardiotoxicity, hepatic, or renal dysfunction.

### 2.3. Treatment regimens

#### 2.3.1. Control group: AP regimen

Chemotherapy cycle: every 3 weeks (21 days) was defined as 1 cycle.

Dosing schedule: doxorubicin, 25 mg/m²/d, intravenous infusion, administered for 3 consecutive days (days 1–3). Cisplatin, 100 mg/m², intravenous infusion on day 1 (with adequate hydration and diuresis to protect renal function).

Treatment duration: patients continued treatment until one of the following occurred: disease progression; intolerable toxicity; withdrawal of informed consent; or investigator’s judgment that treatment should be discontinued.

#### 2.3.2. Exposure group: Paz + AP regimen

Chemotherapy cycle: every 3 weeks (21 days) was defined as 1 cycle.

Dosing schedule: pazopanib, 800 mg, orally once daily on an empty stomach (1 hour before or 2 hours after meals). It was administered continuously from day 4 to day 21 of each cycle. Pazopanib was withheld during chemotherapy administration (days 1–3), theoretically to reduce overlapping toxicities (especially myelosuppression and mucositis), while maintaining vascular normalization for most of the cycle, potentially enhancing chemotherapy delivery and efficacy. Doxorubicin and cisplatin: same dosage and administration schedule as in the control group (doxorubicin on days 1–3 and cisplatin on day 1).

Treatment duration: identical to the control group, continued until disease progression, intolerable toxicity, or patient withdrawal.

#### 2.3.3. Supportive care and monitoring

All patients in both groups received standard supportive treatment, including antiemetics, hydration, diuretics, and hepatoprotective agents, to reduce chemotherapy-related toxicities. During treatment, complete blood counts, liver and renal function tests, electrolytes, and echocardiography were closely monitored, and dose adjustments or treatment delays were made according to the severity of AEs.

### 2.4. Data collection

#### 2.4.1. Collection of general clinical data

Baseline demographic information was collected, including age, sex, and performance status (ECOG score). Tumor-related characteristics were also recorded, including the primary site (femur, tibia, or other locations), recurrence type (local, distant metastasis – primarily lung, or local with distant metastasis), and recurrence-free interval (RFI). In addition, prior treatment history was summarized, including whether patients underwent radical surgery, radiotherapy, and first-line chemotherapy regimens (MAP or MAP + IFO).

#### 2.4.2. Collection of efficacy data

Radiological response was assessed in all patients according to response evaluation criteria in solid tumors (RECIST 1.1) criteria, and categorized as complete response (CR), partial response (PR), stable disease, or progressive disease. Objective response rate (ORR = CR + PR) and disease control rate (DCR = CR + PR + SD) were calculated and compared between the 2 groups.

Radiologic evaluations were independently performed by 2 experienced radiologists who were blinded to the treatment regimen. In cases of discrepancy, consensus was reached through discussion or adjudication by a third senior radiologist.

#### 2.4.3. Collection of survival outcomes

PFS and OS were recorded. PFS was defined as the time from initiation of treatment after recurrence to radiological disease progression or death. OS was defined as the time from initiation of treatment after recurrence to death from any cause. All patients were followed until disease progression or death, with a maximum follow-up duration of 24 months.

#### 2.4.4. Collection of safety and compliance data

Adverse events (AEs) observed during treatment were recorded for both groups, including hematologic toxicities (neutropenia, anemia, thrombocytopenia) and non-hematologic toxicities (hepatic dysfunction, renal dysfunction, hypertension, hand-foot syndrome, etc). In addition, ≥grade 3 AEs, serious AEs, and treatment-related deaths were documented. Compliance data included cumulative doses of doxorubicin and cisplatin, relative dose intensity (RDI ≥ 80%), as well as dose reductions and discontinuation of pazopanib.

### 2.5. Statistical analysis

Data analyses were performed using SPSS version 26.0 (IBM Corporation, Armonk) and R version 4.2.2. Continuous variables (such as age and RFI) were expressed as mean ± standard deviation or median (interquartile range), and compared between groups using the *t*-test or the rank-sum test. Categorical variables (such as sex and ECOG score) were compared using the χ² test or Fisher’s exact test. To minimize confounding factors, PSM was applied with a 1:1 matching ratio and a caliper width of 0.2. Covariates included age, sex, ECOG score, recurrence site, prior treatments, and RFI, and balance was evaluated using standardized mean differences. Treatment efficacy was assessed according to RECIST 1.1 criteria, with ORR and DCR compared between groups. Survival outcomes were analyzed using the Kaplan–Meier method to generate survival curves, with intergroup comparisons performed by the log-rank test. Hazard ratios and 95% confidence intervals (CIs) were calculated using Cox regression, and subgroup analyses were conducted. All statistical tests were 2-sided, and a *P*-value < .05 was considered statistically significant.

## 3. Results

### 3.1. Comparison of baseline characteristics between the 2 groups

A total of 100 patients were included, with 50 in the exposure group and 50 in the control group. There were no statistically significant differences between the 2 groups in terms of age (23.4 ± 6.8 years vs 22.9 ± 7.2 years), sex distribution, or ECOG performance status (with the majority scoring 0–1, accounting for over 80%), as shown in Table 1. The primary tumor sites were mainly the femur and tibia, while pulmonary metastasis was the most common type of recurrence (approximately two-thirds of cases), with similar proportions between the groups. The median RFI was 8 months in the exposure group and 9 months in the control group. All patients had undergone radical surgery; approximately 60% received the MAP chemotherapy regimen, while the remainder received MAP + IFO. About 1-quarter of patients had received radiotherapy. Overall, the 2 groups were well balanced in key demographic and disease characteristics, providing good comparability.

**Table 1:** Baseline characteristics of patients in the research and control groups.

Characteristic	Exposure group (n = 50)	Control group (n = 50)	*P* value	SMD
Age, yr, mean ± SD	23.4 ± 6.8	22.9 ± 7.2	.74	0.07
Sex, n (%)			.83	0.05
Male	31 (62.0%)	30 (60.0%)		
Female	19 (38.0%)	20 (40.0%)		
ECOG performance status, n (%)			.66	0.09
0–1	42 (84.0%)	40 (80.0%)		
2	8 (16.0%)	10 (20.0%)		
Primary site, n (%)			.89	0.08
Femur	24 (48.0%)	23 (46.0%)		
Tibia	15 (30.0%)	14 (28.0%)		
Others (humerus/pelvis, etc)	11 (22.0%)	13 (26.0%)		
Type of recurrence, n (%)			.71	0.06
Local recurrence	9 (18.0%)	11 (22.0%)		
Distant metastasis (mainly lung)	34 (68.0%)	32 (64.0%)		
Local + distant	7 (14.0%)	7 (14.0%)		
Recurrence-to-retreatment interval (RFI), mo, median (IQR)	8 (5–14)	9 (6–15)	.52	0.12
Previous treatments, n (%)			.77	0.1
Major surgery	50 (100%)	50 (100%)	–	–
Radiotherapy	12 (24.0%)	13 (26.0%)		
First-line chemotherapy (MAP regimen)	29 (58.0%)	28 (56.0%)		
First-line chemotherapy (MAP + IFO regimen)	21 (42.0%)	22 (44.0%)		

ECOG = Eastern Cooperative Oncology Group, RFI = recurrence-to-retreatment interval, SD = standard deviation, SMD = standardized mean differences.

### 3.2. Comparison of tumor response

Tumor response was evaluated according to RECIST 1.1 criteria, as shown in Table 2. Overall, the ORR in the exposure group was 26.0%, which was higher than that in the control group (14.0%), although the difference did not reach statistical significance (*P* = .13). However, the DCR in the exposure group was significantly better than that in the control group (62.0% vs 42.0%, *P* = .046), suggesting that the addition of pazopanib may provide an advantage in delaying disease progression.

**Table 2:** Treatment response according to RECIST 1.1.

Response outcome	Exposure group (n = 50)	Control group (n = 50)	Test statistic	*P* value
CR, n (%)	1 (2.0%)	0 (0.0%)		
PR, n (%)	12 (24.0%)	7 (14.0%)		
SD, n (%)	18 (36.0%)	14 (28.0%)		
PD, n (%)	19 (38.0%)	29 (58.0%)		
ORR (CR + PR), n (%)	13 (26.0%)	7 (14.0%)	χ² = 2.25	.13
DCR (CR + PR + SD), n (%)	31 (62.0%)	21 (42.0%)	χ² = 4.00	.046

CR = complete response, DCR = disease control rate, ORR = objective response rate, PD = progressive disease, PR = partial response, RECIST = response evaluation criteria in solid tumors, SD = stable disease.

### 3.3. Progression-free survival (PFS)

**Figure 1.**
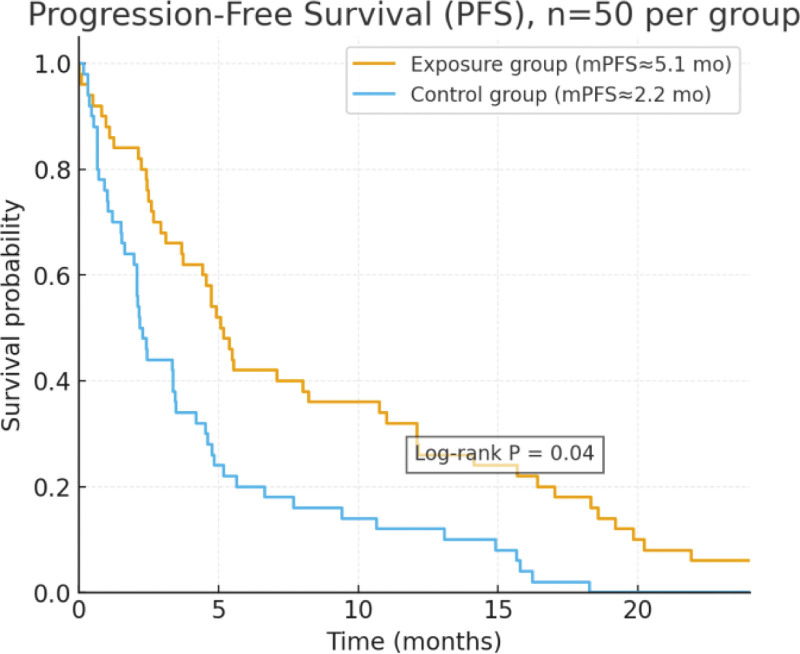
Comparison of progression-free survival (PFS) between the exposed group and the control group. PFS = progression-free survival.

Kaplan–Meier analysis demonstrated that the median PFS was longer in the exposure group compared with the control group (5.1 months vs 2.2 months), as shown in Figure 1. The survival curves of the 2 groups diverged early and remained separated throughout the 24-month follow-up period. The log-rank test indicated that the difference between the groups was statistically significant (*P* = .04).

### 3.4. Overall survival (OS)

**Figure 2.**
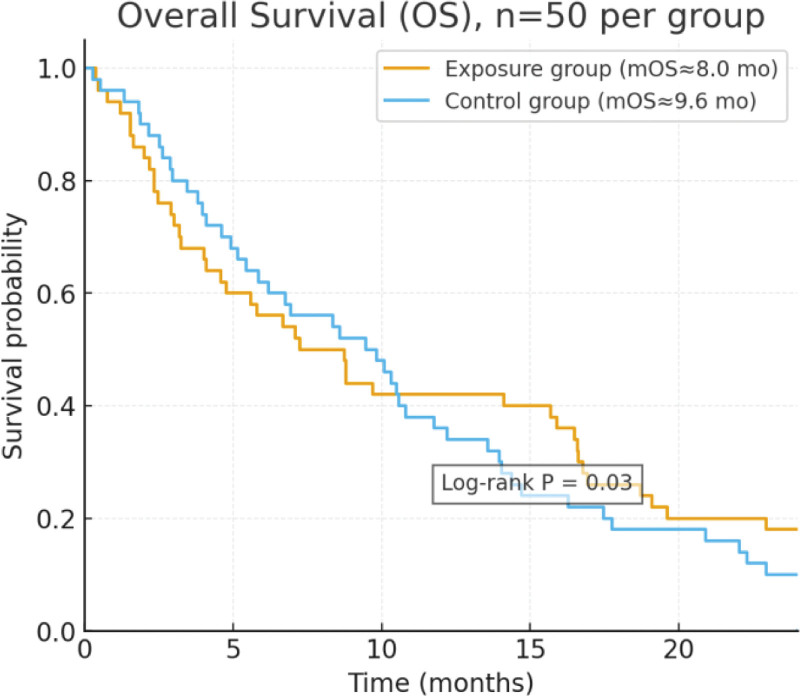
Comparison of overall survival (OS) between the exposed group and the control group. OS = overall survival.

Regarding OS, the median OS was 8.0 months in the exposure group and 9.6 months in the control group, as shown in Figure 2. Although the median OS was slightly longer in the control group, its survival curve declined more rapidly after 12 months of follow-up, whereas the curve for the exposure group showed a relatively slower decline. The log-rank test indicated that the difference between the 2 groups was statistically significant (*P* = .03).

### 3.5. Safety and treatment compliance

Both groups showed a high overall incidence of AEs. Common hematologic toxicities, such as neutropenia, anemia, and thrombocytopenia, did not differ significantly between the 2 groups, as shown in Table 3. In terms of non-hematologic toxicities, however, the incidence of hypertension and hand-foot syndrome was significantly higher in the exposure group compared with the control group (28.0% vs 10.0% and 22.0% vs 4.0%, respectively), with statistically significant differences.

**Table 3:** Safety profile and treatment compliance.

Adverse events (AEs)	Exposure group (n = 50)	Control group (n = 50)	*P* value
Any AE, n (%)	46 (92.0%)	44 (88.0%)	.54
Hematologic toxicity, n (%)			
Neutropenia (all grades)	28 (56.0%)	25 (50.0%)	.56
Anemia (all grades)	22 (44.0%)	21 (42.0%)	.84
Thrombocytopenia (all grades)	15 (30.0%)	14 (28.0%)	.82
Non-hematologic toxicity, n (%)			
Hepatic dysfunction(ALT/AST↑)	12 (24.0%)	8 (16.0%)	.32
Renal impairment (Cr↑)	10 (20.0%)	9 (18.0%)	.8
Hypertension	14 (28.0%)	5 (10.0%)	.03
Hand-foot syndrome	11 (22.0%)	2 (4.0%)	.01
Grade ≥ 3 AEs, n (%)	21 (42.0%)	17 (34.0%)	.41
Serious adverse events (SAE), n (%)	5 (10.0%)	4 (8.0%)	.73
Treatment-related death (TRD), n (%)	0 (0.0%)	1 (2.0%)	.32
Treatment compliance			
Doxorubicin cumulative dose ≥ 450 mg/m², n (%)	8 (16.0%)	6 (12.0%)	.57
Cisplatin cumulative dose ≥ 500 mg/m², n (%)	10 (20.0%)	9 (18.0%)	.8
RDI ≥ 80%, n (%)	41 (82.0%)	39 (78.0%)	.63
Pazopanib dose reduction, n (%)	15 (30.0%)	–	–
Pazopanib discontinuation, n (%)	6 (12.0%)	–	–

AE = adverse event, SAE = serious adverse event, TRD = treatment-related death.

The incidence of ≥grade 3 AEs, serious AEs, and treatment-related deaths was comparable between groups. Most patients were able to maintain a relatively high relative dose intensity (RDI ≥ 80%). Approximately one-third of patients in the exposure group required dose reductions due to toxicity, and a few discontinued pazopanib. Overall, the toxicity profile of the combination regimen was manageable, and treatment compliance was favorable.

### 3.6. Subgroup analysis

**Figure 3.**
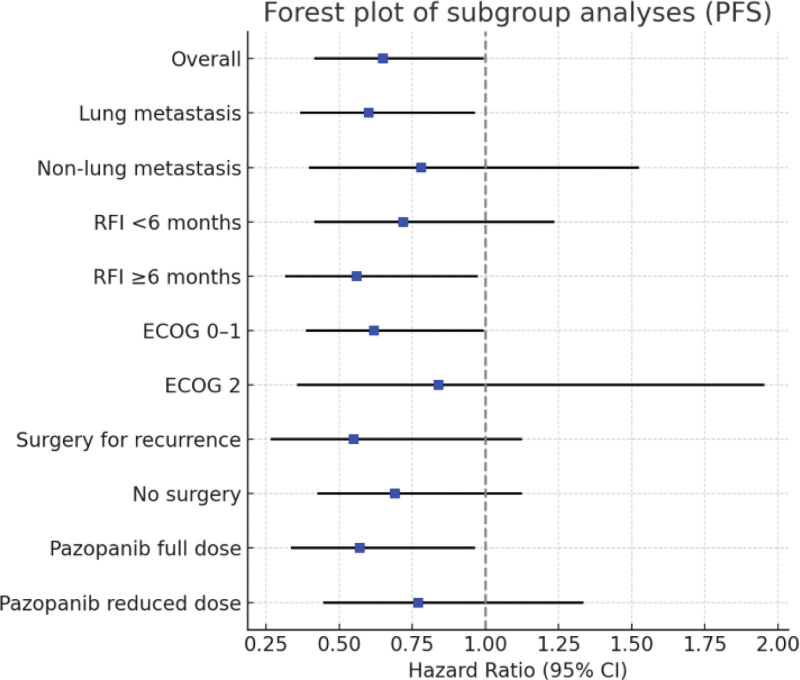
Forest plot of subgroup analysis (PFS). CI = confidence interval, ECOG = Eastern Cooperative Oncology Group, PFS = progression-free survival, RFI = recurrence-free interval.

Overall analysis showed that the exposure group had superior PFS compared with the control group (hazard ratio [HR] = 0.65, 95% CI: 0.42–0.99), as presented in Table 4. In subgroup analyses, patients with pulmonary metastases (HR = 0.60, 95% CI: 0.37–0.96), recurrence-to-retreatment interval ≥6 months (HR = 0.56, 95% CI: 0.32–0.97), and ECOG performance status 0 to 1 (HR = 0.62, 95% CI: 0.39–0.99) all demonstrated significant benefit, as shown in Table 4 and Figure 3.

**Table 4:** Subgroup analyses of progression-free survival (PFS, exploratory).

Subgroup	No. of patients	Events	HR (95% CI)	*P*_interaction_
	Research/control	Research/control		
Overall	50/50	46/48	**0.65 (0.42–0.99**)	–
Site of recurrence				.21
Lung metastasis	34/32	31/31	**0.60 (0.37–0.96**)	
Non-lung (local/other distant)	16/18	15/17	0.78 (0.40–1.52)	
Recurrence-to-treatment interval (RFI)				.18
<6 mo	22/21	21/21	0.72 (0.42–1.23)	
≥6 mo	28/29	25/27	**0.56 (0.32–0.97**)	
ECOG performance status				.34
0–1	42/40	38/38	**0.62 (0.39–0.99**)	
2	8/10	8/10	0.84 (0.36–1.95)	
Surgery for recurrent lesion				.27
Yes	14/13	12/12	0.55 (0.27–1.12)	
No	36/37	34/36	0.69 (0.43–1.12)	
Pazopanib dose (research subgroup vs control)	–	–	–	.12
Full dose (≥800 mg)	29/50	27/48	**0.57 (0.34–0.96**)	
Reduced dose (<800 mg)	21/50	19/48	0.77 (0.45–1.33)	

Bold value indicates HR values with *P* < .05.

CI = confidence interval, ECOG = Eastern Cooperative Oncology Group, HR = hazard ratio, PFS = progression-free survival, RFI = recurrence-to-retreatment interval.

For patients with non-pulmonary recurrence, RFI < 6 months, ECOG score of 2, or those who did not undergo surgery for recurrent lesions, the differences were not statistically significant, although the overall trend still favored the exposure group. Notably, within the exposure group, patients who maintained full-dose pazopanib treatment derived more pronounced benefit (HR = 0.57, 95% CI: 0.34–0.96), whereas no clear benefit was observed in those who required dose reduction. Tests for interaction across subgroups were not statistically significant, suggesting a generally consistent treatment effect, with certain subgroups (pulmonary metastases, longer RFI, better performance status, and full-dose pazopanib) potentially deriving greater benefit.

## 4. Discussion

In this study, we compared the efficacy and safety of pazopanib combined with doxorubicin and cisplatin versus conventional chemotherapy in patients with recurrent osteosarcoma. The results demonstrated that the combination regimen provided certain advantages in DCR and PFS, while overall AEs remained manageable. These findings suggest that pazopanib may serve as a complementary component in the comprehensive management of recurrent osteosarcoma, although further investigation is warranted to clarify its mechanisms and identify suitable patient populations.

First, the DCR in the exposure group (62.0%) was significantly higher than that in the control group (42.0%), and median PFS was prolonged to 5.1 months, indicating that the addition of pazopanib may help delay disease progression. The underlying mechanism may be related to the anti-angiogenic effects of pazopanib. By inhibiting vascular endothelial growth factor receptor and PDGFR, pazopanib may improve the intratumoral delivery of chemotherapeutic agents during the “vascular normalization” phase, thereby enhancing the cytotoxic efficacy of chemotherapy.^[14,15]^ This mechanism has been partially validated in studies of soft tissue sarcomas and other malignancies, suggesting that a similar effect may exist in osteosarcoma as well.

However, the results regarding OS were more complex. Although the median OS in the control group (9.6 months) was slightly longer than that in the exposure group (8.0 months), the survival curve of the control group declined more steeply after 12 months of follow-up, whereas the exposure group maintained a relatively slower decline. This may reflect 2 possible scenarios: on the 1 hand, long-term maintenance with pazopanib could delay late-stage disease progression in some patients; on the other hand, drug-related toxicities in the exposure group might have affected treatment continuity during the earlier stages.^[16,17]^ Notably, subgroup analysis indicated that patients with pulmonary metastases, recurrence-to-retreatment interval ≥6 months, and good performance status derived more significant benefits in terms of PFS, suggesting that these populations may represent more appropriate candidates for pazopanib-based therapy in future clinical practice.^[18,19]^

In terms of safety, the overall toxicity of the combination therapy was manageable, but certain pazopanib-related AEs were clearly observed, such as a significantly increased incidence of hypertension and hand-foot syndrome. This is consistent with its mechanism of action, whereby inhibition of the VEGF pathway alters endothelial function and leads to elevated blood pressure. These findings highlight the need for careful monitoring and timely supportive management in clinical practice to ensure that patients can maintain adequate dose intensity.^[20,21]^ Importantly, within the exposure group, patients who were able to sustain full-dose pazopanib treatment derived the most significant benefit, whereas those requiring dose reductions showed less evident effects, further underscoring the importance of dose adherence.

## 5. Limitations and future directions

This study has several limitations that should be acknowledged. First, as a single-center retrospective cohort study with a relatively small sample size, inherent selection bias and confounding factors cannot be completely eliminated, even after PSM. Second, although the imaging assessments were conducted independently by blinded radiologists, potential inter-observer variability may still exist. Third, the follow-up duration was limited, which may have affected the evaluation of long-term OS outcomes. Additionally, the absence of biomarker analysis limited our ability to identify potential predictive factors for pazopanib efficacy and toxicity. Future research should focus on conducting multicenter prospective randomized controlled trials to validate the clinical benefit of pazopanib in combination with chemotherapy for recurrent osteosarcoma. Furthermore, translational studies exploring molecular biomarkers, angiogenic signatures, and pharmacogenomic predictors may help identify patient subgroups most likely to benefit from this regimen. Integration of real-world data and novel imaging or circulating biomarkers could also enhance understanding of treatment response dynamics and optimize individualized therapeutic strategies.

Overall, this study suggests that pazopanib combined with chemotherapy may improve disease control and prolong PFS in patients with recurrent osteosarcoma, with particularly pronounced benefits observed in selected subgroups. However, its impact on OS remains inconclusive, potentially influenced by sample size, follow-up duration, and subsequent treatments. Moreover, as this was a retrospective study, prospective randomized controlled trials are still needed to validate these findings and to explore optimized dosing strategies that balance efficacy and safety.

## Author contributions

**Conceptualization:** Wei-Luo Cai, Zheng-Wang Sun, Meng Fang, Wang-Jun Yan, Xiong-Sheng Chen, Gen-Long Jiao.

**Data curation:** Wei-Luo Cai, Mo Cheng, Zheng-Wang Sun, Meng Fang, Wang-Jun Yan, Xiong-Sheng Chen, Gen-Long Jiao.

**Formal analysis:** Wei-Luo Cai, Mo Cheng, Zheng-Wang Sun, Meng Fang, Gen-Long Jiao.

**Funding acquisition:** Mo Cheng, Zheng-Wang Sun, Xiong-Sheng Chen, Gen-Long Jiao.

**Investigation:** Zheng-Wang Sun, Wang-Jun Yan.

**Writing** – **original draft:** Wei-Luo Cai, Meng Fang, Xiong-Sheng Chen, Gen-Long Jiao.

**Writing** – **review & editing:** Wei-Luo Cai, Meng Fang, Wang-Jun Yan, Xiong-Sheng Chen, Gen-Long Jiao.
